# 7-Chloro­indoline-2,3-dione

**DOI:** 10.1107/S1600536809051526

**Published:** 2009-12-04

**Authors:** Jie Sun, Zai-Sheng Cai

**Affiliations:** a College of Food Science and Light Industry, Nanjing University of Technology, Xinmofan Road No. 5 Nanjing, Nanjing 210009, People’s Republic of China; b College of Chemistry, Chemical Engineering and Biotechnology, Donghua University, North Renmin Road No. 2999 Songjiang, Shanghai 201620, People’s Republic of China

## Abstract

There are two mol­ecules in the asymmetric unit of the title compound, C_8_H_4_ClNO_2_. In the crystal, they are linked by N—H⋯O hydrogen bonds, generating centrosymmetric, tetra­meric assemblies. A C—H⋯O inter­action also occurs.

## Related literature

For general background to oxyphenastatin derivatives and further synthetic details, see: Uddin *et al.* (2007 ▶). For bond-length data, see: Allen *et al.* (1987 ▶).
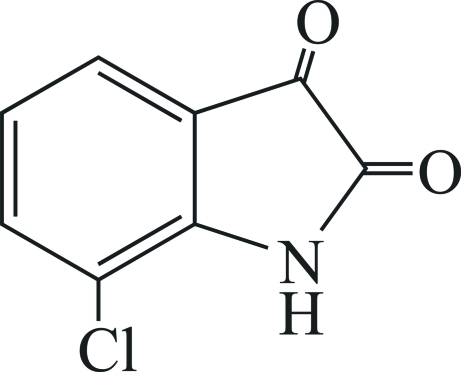


## Experimental

### 

#### Crystal data

C_8_H_4_ClNO_2_
*M* *_r_* = 181.57
Triclinic, 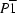

*a* = 7.2450 (14) Å
*b* = 8.6080 (17) Å
*c* = 12.470 (3) Å
α = 86.95 (3)°
β = 78.02 (3)°
γ = 84.89 (3)°
*V* = 757.2 (3) Å^3^
*Z* = 4
Mo *K*α radiation
μ = 0.45 mm^−1^
*T* = 293 K
0.30 × 0.20 × 0.10 mm

#### Data collection

Enraf–Nonius CAD-4 diffractometer
Absorption correction: ψ scan (North *et al.*, 1968 ▶) *T*_min_ = 0.876, *T*_max_ = 0.956
2988 measured reflections
2749 independent reflections
2051 reflections with *I* > 2σ(*I*)
*R*_int_ = 0.047
3 standard reflections every 200 reflections
intensity decay: 1%

#### Refinement

*R*[*F* ^2^ > 2σ(*F* ^2^)] = 0.055
*wR*(*F* ^2^) = 0.160
*S* = 1.01
2749 reflections
217 parameters
13 restraints
H-atom parameters constrained
Δρ_max_ = 0.63 e Å^−3^
Δρ_min_ = −0.29 e Å^−3^

### 

Data collection: *CAD-4 Software* (Enraf–Nonius, 1989 ▶); cell refinement: *CAD-4 Software*; data reduction: *XCAD4* (Harms & Wocadlo,1995 ▶); program(s) used to solve structure: *SHELXS97* (Sheldrick, 2008 ▶); program(s) used to refine structure: *SHELXL97* (Sheldrick, 2008 ▶); molecular graphics: *PLATON* (Spek, 2009 ▶); software used to prepare material for publication: *SHELXL97* and *PLATON* (Spek, 2009 ▶).

## Supplementary Material

Crystal structure: contains datablocks global, I. DOI: 10.1107/S1600536809051526/hb5233sup1.cif

Structure factors: contains datablocks I. DOI: 10.1107/S1600536809051526/hb5233Isup2.hkl

Additional supplementary materials:  crystallographic information; 3D view; checkCIF report

## Figures and Tables

**Table 1:** Hydrogen-bond geometry (Å, °)

*D*—H⋯*A*	*D*—H	H⋯*A*	*D*⋯*A*	*D*—H⋯*A*
N1—H1*A*⋯O4^i^	0.86	2.12	2.961 (4)	165
N2—H2*B*⋯O4^i^	0.86	2.10	2.923 (3)	160
C14—H14*A*⋯O2^ii^	0.93	2.46	3.385 (4)	172

Symmetry codes: (i) 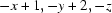; (ii) 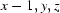.

**Fig. 1.**
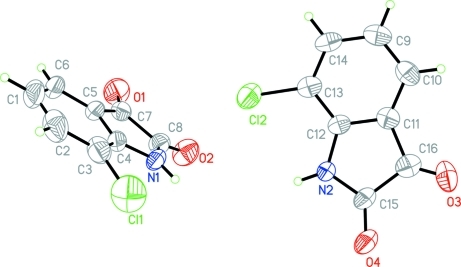
The molecular structure of (I) with displacement ellipsoids drawn at the 50% probability level.

**Fig. 2.**
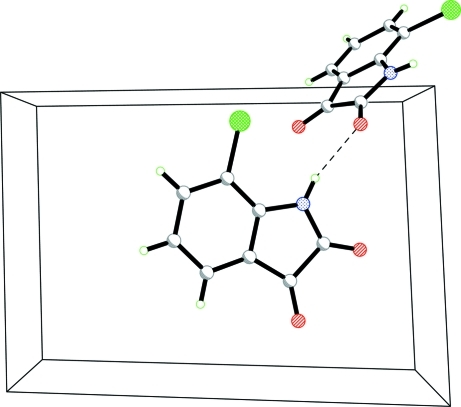
A partial packing diagram of (I). Hydrogen bonds are shown as dashed lines.

## supplementary crystallographic information

### Experimental

85 g (0.06 mol) Sodium sulfate and 300 ml water was added into a 1000 ml three mouthed flask, mixed till the sodium sulfate dissolved, then a saturated solution of 18 g (0.11 mol) chloral hydrate was added. While stirring, the mixture of 12.7 g (0.1 mol) *p*-chloroaniline, 12 ml hydrochloric acid and 100 ml water was dropped to the reaction mixture causing white precipitation. Then 22 g (0.32 mol) hydroxylamine hydrochloride was added and the mixture was heated to 348 K. After 5 h, light yellow precipitation appeared, filtered and washed with water, dried and then added the yellow precipitation into concentrated sulfuric acid (50 ml) in batches at 353 K. Heated to 363 K and stirred for 30 minutes and dumped the mixture into ice water (1000 ml), stirred for 40 minutes, filtered and washed with water to neutral, dried and Yellow blocks of (I) were obtained by slow evaporation of an acetone solution (yield; 90%, m.p. 463 K).

### Refinement

H atoms were positioned geometrically, with N—H = 0.86Å and C—H = 0.93Å and refined as riding with *U*_iso_(H) = *xU*_eq_(C,*O*,*N*), where *x* = 1.5 for NH H and *x* = 1.2 for all other H atoms.

### Crystal data

**Table:**

C_8_H_4_ClNO_2_	*Z* = 4
*M**_r_* = 181.57	*F*(000) = 368
Triclinic, *P*1	*D*_x_ = 1.593 Mg m^−^^3^
Hall symbol: -P 1	Melting point: 463K K
*a* = 7.2450 (14) Å	Mo *K*α radiation, λ = 0.71073 Å
*b* = 8.6080 (17) Å	Cell parameters from 25 reflections
*c* = 12.470 (3) Å	θ = 9–13°
α = 86.95 (3)°	µ = 0.45 mm^−^^1^
β = 78.02 (3)°	*T* = 293 K
γ = 84.89 (3)°	Block, yellow
*V* = 757.2 (3) Å^3^	0.30 × 0.20 × 0.10 mm

### Data collection

**Table:**

Enraf–Nonius CAD-4 diffractometer	2051 reflections with *I* > 2σ(*I*)
Radiation source: fine-focus sealed tube	*R*_int_ = 0.047
graphite	θ_max_ = 25.3°, θ_min_ = 1.7°
ω/2θ scans	*h* = 0→8
Absorption correction: ψ scan (North *et al.*, 1968)	*k* = −10→10
*T*_min_ = 0.876, *T*_max_ = 0.956	*l* = −14→14
2988 measured reflections	3 standard reflections every 200 reflections
2749 independent reflections	intensity decay: 1%

### Refinement

**Table:**

Refinement on *F*^2^	Primary atom site location: structure-invariant direct methods
Least-squares matrix: full	Secondary atom site location: difference Fourier map
*R*[*F*^2^ > 2σ(*F*^2^)] = 0.055	Hydrogen site location: inferred from neighbouring sites
*wR*(*F*^2^) = 0.160	H-atom parameters constrained
*S* = 1.01	*w* = 1/[σ^2^(*F*_o_^2^) + (0.1*P*)^2^ + 0.250*P*] where *P* = (*F*_o_^2^ + 2*F*_c_^2^)/3
2749 reflections	(Δ/σ)_max_ < 0.001
217 parameters	Δρ_max_ = 0.63 e Å^−^^3^
13 restraints	Δρ_min_ = −0.29 e Å^−^^3^

### Special details

**Table:**

Geometry. All e.s.d.'s (except the e.s.d. in the dihedral angle between two l.s. planes) are estimated using the full covariance matrix. The cell e.s.d.'s are taken into account individually in the estimation of e.s.d.'s in distances, angles and torsion angles; correlations between e.s.d.'s in cell parameters are only used when they are defined by crystal symmetry. An approximate (isotropic) treatment of cell e.s.d.'s is used for estimating e.s.d.'s involving l.s. planes.
Refinement. Refinement of *F*^2^ against ALL reflections. The weighted *R*-factor *wR* and goodness of fit *S* are based on *F*^2^, conventional *R*-factors *R* are based on *F*, with *F* set to zero for negative *F*^2^. The threshold expression of *F*^2^ > σ(*F*^2^) is used only for calculating *R*-factors(gt) *etc*. and is not relevant to the choice of reflections for refinement. *R*-factors based on *F*^2^ are statistically about twice as large as those based on *F*, and *R*- factors based on ALL data will be even larger.

### Fractional atomic coordinates and isotropic or equivalent isotropic displacement parameters (Å^2^)

**Table:**

	*x*	*y*	*z*	*U*_iso_*/*U*_eq_	
Cl1	0.28644 (18)	0.90528 (12)	0.43418 (10)	0.0839 (4)	
N1	0.4403 (4)	0.6203 (3)	0.28727 (19)	0.0415 (6)	
H1A	0.4599	0.7099	0.2550	0.050*	
O1	0.4519 (4)	0.2163 (3)	0.3096 (2)	0.0731 (8)	
O2	0.5909 (4)	0.4581 (3)	0.1488 (2)	0.0662 (7)	
C1	0.1629 (6)	0.4991 (7)	0.6003 (3)	0.0780 (13)	
H1B	0.1019	0.4674	0.6702	0.094*	
C2	0.1735 (5)	0.6597 (6)	0.5739 (3)	0.0701 (11)	
H2A	0.1201	0.7328	0.6261	0.084*	
C3	0.2638 (5)	0.7090 (4)	0.4697 (3)	0.0541 (9)	
C4	0.3423 (4)	0.5985 (4)	0.3954 (2)	0.0393 (7)	
C5	0.3300 (4)	0.4400 (4)	0.4231 (3)	0.0462 (8)	
C6	0.2399 (5)	0.3892 (5)	0.5258 (3)	0.0636 (11)	
H6A	0.2321	0.2834	0.5435	0.076*	
C7	0.4285 (5)	0.3544 (4)	0.3265 (3)	0.0472 (8)	
C8	0.5004 (4)	0.4814 (4)	0.2399 (3)	0.0441 (7)	
Cl2	0.12910 (13)	0.66786 (10)	0.12400 (7)	0.0589 (3)	
N2	0.3136 (3)	0.9103 (3)	−0.04844 (19)	0.0353 (5)	
H2B	0.3737	0.8832	0.0031	0.042*	
O3	0.2217 (4)	1.0991 (3)	−0.2813 (2)	0.0618 (7)	
O4	0.4995 (3)	1.1009 (3)	−0.13853 (18)	0.0533 (6)	
C9	−0.1833 (5)	0.7602 (4)	−0.1086 (3)	0.0486 (8)	
H9A	−0.2948	0.7290	−0.1235	0.058*	
C10	−0.0841 (4)	0.8681 (4)	−0.1775 (3)	0.0472 (8)	
H10A	−0.1282	0.9111	−0.2384	0.057*	
C11	0.0833 (4)	0.9115 (3)	−0.1544 (2)	0.0403 (7)	
C12	0.1483 (4)	0.8492 (3)	−0.0617 (2)	0.0352 (6)	
C13	0.0469 (4)	0.7416 (3)	0.0075 (2)	0.0414 (7)	
C14	−0.1181 (4)	0.6972 (4)	−0.0167 (3)	0.0446 (7)	
H14A	−0.1866	0.6243	0.0290	0.053*	
C15	0.3646 (4)	1.0178 (4)	−0.1286 (2)	0.0438 (7)	
C16	0.2210 (4)	1.0242 (4)	−0.2053 (3)	0.0473 (8)	

### Atomic displacement parameters (Å^2^)

**Table:**

	*U*^11^	*U*^22^	*U*^33^	*U*^12^	*U*^13^	*U*^23^
Cl1	0.1048 (9)	0.0609 (6)	0.0837 (8)	0.0087 (6)	−0.0158 (6)	−0.0213 (5)
N1	0.0483 (14)	0.0400 (13)	0.0346 (13)	−0.0113 (11)	−0.0040 (11)	0.0076 (10)
O1	0.102 (2)	0.0405 (14)	0.083 (2)	−0.0138 (13)	−0.0326 (17)	0.0086 (13)
O2	0.0718 (17)	0.0661 (16)	0.0523 (15)	−0.0055 (13)	0.0086 (13)	−0.0098 (12)
C1	0.049 (2)	0.144 (4)	0.040 (2)	−0.025 (2)	−0.0059 (17)	0.025 (2)
C2	0.050 (2)	0.116 (3)	0.0408 (19)	0.003 (2)	−0.0039 (16)	−0.010 (2)
C3	0.0449 (18)	0.073 (2)	0.0444 (18)	−0.0010 (16)	−0.0110 (15)	−0.0040 (16)
C4	0.0342 (15)	0.0512 (17)	0.0333 (15)	−0.0076 (12)	−0.0091 (12)	0.0059 (13)
C5	0.0420 (16)	0.0554 (19)	0.0443 (17)	−0.0175 (14)	−0.0149 (14)	0.0180 (14)
C6	0.054 (2)	0.092 (3)	0.049 (2)	−0.029 (2)	−0.0186 (18)	0.033 (2)
C7	0.0547 (19)	0.0435 (18)	0.0501 (19)	−0.0152 (14)	−0.0237 (16)	0.0084 (14)
C8	0.0424 (17)	0.0451 (17)	0.0440 (18)	−0.0062 (13)	−0.0062 (14)	0.0011 (13)
Cl2	0.0661 (6)	0.0547 (5)	0.0548 (5)	−0.0123 (4)	−0.0093 (4)	0.0095 (4)
N2	0.0392 (13)	0.0354 (12)	0.0323 (12)	−0.0086 (10)	−0.0088 (10)	0.0054 (10)
O3	0.0607 (15)	0.0807 (18)	0.0447 (14)	0.0069 (13)	−0.0190 (12)	0.0044 (13)
O4	0.0564 (14)	0.0499 (13)	0.0496 (13)	−0.0119 (11)	−0.0020 (11)	0.0135 (10)
C9	0.0388 (16)	0.0509 (18)	0.057 (2)	−0.0076 (14)	−0.0067 (15)	−0.0162 (16)
C10	0.0406 (17)	0.0563 (19)	0.0460 (18)	0.0031 (15)	−0.0118 (14)	−0.0118 (15)
C11	0.0371 (15)	0.0433 (16)	0.0394 (16)	−0.0018 (12)	−0.0046 (13)	−0.0074 (13)
C12	0.0326 (14)	0.0329 (14)	0.0371 (15)	−0.0030 (11)	0.0010 (12)	−0.0052 (11)
C13	0.0405 (16)	0.0378 (15)	0.0429 (17)	−0.0050 (12)	0.0005 (13)	−0.0080 (13)
C14	0.0376 (16)	0.0398 (16)	0.0521 (18)	−0.0101 (12)	0.0046 (14)	−0.0058 (13)
C15	0.0442 (17)	0.0437 (16)	0.0406 (16)	−0.0105 (14)	−0.0012 (14)	0.0070 (13)
C16	0.0441 (18)	0.0392 (16)	0.055 (2)	−0.0014 (13)	−0.0017 (15)	−0.0040 (15)

### Geometric parameters (Å, °)

**Table:**

Cl1—C3	1.737 (4)	Cl2—C13	1.749 (3)
N1—C8	1.358 (4)	N2—C15	1.342 (4)
N1—C4	1.399 (4)	N2—C12	1.393 (4)
N1—H1A	0.8600	N2—H2B	0.8600
O1—C7	1.210 (4)	O3—C16	1.116 (4)
O2—C8	1.205 (4)	O4—C15	1.244 (4)
C1—C6	1.362 (6)	C9—C10	1.377 (5)
C1—C2	1.410 (7)	C9—C14	1.394 (5)
C1—H1B	0.9300	C9—H9A	0.9300
C2—C3	1.391 (5)	C10—C11	1.389 (4)
C2—H2A	0.9300	C10—H10A	0.9300
C3—C4	1.366 (5)	C11—C12	1.401 (4)
C4—C5	1.396 (4)	C11—C16	1.478 (4)
C5—C6	1.379 (5)	C12—C13	1.384 (4)
C5—C7	1.465 (5)	C13—C14	1.382 (4)
C6—H6A	0.9300	C14—H14A	0.9300
C7—C8	1.541 (4)	C15—C16	1.548 (5)
			
C8—N1—C4	111.1 (2)	C15—N2—C12	109.5 (2)
C8—N1—H1A	124.5	C15—N2—H2B	125.2
C4—N1—H1A	124.5	C12—N2—H2B	125.2
C6—C1—C2	121.4 (3)	C10—C9—C14	120.5 (3)
C6—C1—H1B	119.3	C10—C9—H9A	119.7
C2—C1—H1B	119.3	C14—C9—H9A	119.7
C3—C2—C1	120.0 (4)	C9—C10—C11	118.7 (3)
C3—C2—H2A	120.0	C9—C10—H10A	120.6
C1—C2—H2A	120.0	C11—C10—H10A	120.6
C4—C3—C2	118.4 (4)	C10—C11—C12	120.8 (3)
C4—C3—Cl1	119.7 (3)	C10—C11—C16	134.1 (3)
C2—C3—Cl1	121.8 (3)	C12—C11—C16	105.0 (3)
C3—C4—C5	120.7 (3)	C13—C12—N2	126.7 (3)
C3—C4—N1	128.4 (3)	C13—C12—C11	120.1 (3)
C5—C4—N1	110.9 (3)	N2—C12—C11	113.2 (2)
C6—C5—C4	121.6 (4)	C14—C13—C12	118.9 (3)
C6—C5—C7	131.6 (3)	C14—C13—Cl2	121.9 (2)
C4—C5—C7	106.8 (3)	C12—C13—Cl2	119.2 (2)
C1—C6—C5	117.9 (4)	C13—C14—C9	121.0 (3)
C1—C6—H6A	121.1	C13—C14—H14A	119.5
C5—C6—H6A	121.1	C9—C14—H14A	119.5
O1—C7—C5	131.5 (3)	O4—C15—N2	126.6 (3)
O1—C7—C8	123.4 (3)	O4—C15—C16	125.7 (3)
C5—C7—C8	105.0 (3)	N2—C15—C16	107.7 (3)
O2—C8—N1	128.3 (3)	O3—C16—C11	128.3 (3)
O2—C8—C7	125.5 (3)	O3—C16—C15	127.2 (3)
N1—C8—C7	106.2 (3)	C11—C16—C15	104.5 (3)
			
C6—C1—C2—C3	0.1 (6)	C14—C9—C10—C11	0.8 (5)
C1—C2—C3—C4	−0.7 (5)	C9—C10—C11—C12	−1.3 (4)
C1—C2—C3—Cl1	−178.2 (3)	C9—C10—C11—C16	−177.5 (3)
C2—C3—C4—C5	0.8 (5)	C15—N2—C12—C13	−176.5 (3)
Cl1—C3—C4—C5	178.4 (2)	C15—N2—C12—C11	1.6 (3)
C2—C3—C4—N1	−179.5 (3)	C10—C11—C12—C13	0.8 (4)
Cl1—C3—C4—N1	−1.9 (5)	C16—C11—C12—C13	178.0 (2)
C8—N1—C4—C3	178.8 (3)	C10—C11—C12—N2	−177.4 (2)
C8—N1—C4—C5	−1.5 (4)	C16—C11—C12—N2	−0.2 (3)
C3—C4—C5—C6	−0.4 (5)	N2—C12—C13—C14	178.0 (3)
N1—C4—C5—C6	179.8 (3)	C11—C12—C13—C14	0.1 (4)
C3—C4—C5—C7	−179.6 (3)	N2—C12—C13—Cl2	−1.0 (4)
N1—C4—C5—C7	0.6 (3)	C11—C12—C13—Cl2	−179.0 (2)
C2—C1—C6—C5	0.3 (6)	C12—C13—C14—C9	−0.6 (4)
C4—C5—C6—C1	−0.1 (5)	Cl2—C13—C14—C9	178.5 (2)
C7—C5—C6—C1	178.8 (3)	C10—C9—C14—C13	0.1 (5)
C6—C5—C7—O1	2.9 (6)	C12—N2—C15—O4	176.1 (3)
C4—C5—C7—O1	−178.1 (4)	C12—N2—C15—C16	−2.2 (3)
C6—C5—C7—C8	−178.7 (3)	C10—C11—C16—O3	−3.6 (6)
C4—C5—C7—C8	0.3 (3)	C12—C11—C16—O3	179.8 (3)
C4—N1—C8—O2	−178.7 (3)	C10—C11—C16—C15	175.6 (3)
C4—N1—C8—C7	1.6 (3)	C12—C11—C16—C15	−1.0 (3)
O1—C7—C8—O2	−2.3 (5)	O4—C15—C16—O3	2.9 (6)
C5—C7—C8—O2	179.1 (3)	N2—C15—C16—O3	−178.8 (3)
O1—C7—C8—N1	177.4 (3)	O4—C15—C16—C11	−176.3 (3)
C5—C7—C8—N1	−1.2 (3)	N2—C15—C16—C11	2.0 (3)

### Hydrogen-bond geometry (Å, °)

**Table:**

*D*—H···*A*	*D*—H	H···*A*	*D*···*A*	*D*—H···*A*
N1—H1A···O4^i^	0.86	2.12	2.961 (4)	165
N2—H2B···O4^i^	0.86	2.10	2.923 (3)	160
C14—H14A···O2^ii^	0.93	2.46	3.385 (4)	172

Symmetry codes: (i) −*x*+1, −*y*+2, −*z*; (ii) *x*−1, *y*, *z*.
